# Phase I trial of active specific immunotherapy with autologous dendritic cells pulsed with autologous irradiated tumor stem cells in hepatitis B positive patients with hepatocellular carcinoma

**DOI:** 10.1186/2051-1426-2-S3-P44

**Published:** 2014-11-06

**Authors:** Michael Bayer, Xiaojin Wang, Chengwei Chen, Xiaosong Chen, Qingchun Fu, Jia He, William Cao, Craig Fredrickson, Jessica Cannon, Andrew Cornforth, Gabriel Nistor, Robert Dillman

**Affiliations:** 1 NeoStem Oncology, LLC, Encinitas, United States; 2 Hospital 85 People's Liberation Army of China, Shanghai, Peoples Republic of China; 3 Shanghai Renji Hospital, Shanghai, Peoples Republic of China; 4 Second Military Medical University, Shanghai, Peoples Republic of China; 5 Cellular Biomedicine Group, Palo Alto, United States; 6 California Stem Cell, Inc., Irvine, United States; 7 Neostem Oncology, LLC, Irvine, United States

## Background

Active or chronic infections may be barriers to successful vaccines, and patients with hepatitis typically are excluded from clinical trials testing therapeutic anticancer vaccines. One concern is vaccines may exacerbate the underlying infection. Hepatocellular carcinoma (HCC) often arises in the context of viral hepatitis B (HBV) or hepatitis C (HBC), with or without cirrhosis. HCC is seldom cured by standard therapy because of undetectable micrometastatic disease that is present at diagnosis. Effective non-toxic adjunctive treatments might improve outcomes for HCC patients who are at high risk for recurrence and death despite surgical resection of their primary hepatoma. Active specific immunotherapy (ASI) with autologous dendritic cells loaded with antigens from autologous tumor stem cell lines has been associated with promising long-term survival in metastatic melanoma, but patients with HBV or HCV were ineligible. Therefore a Phase I safety trial in HCC patients with HBV and/or HIV was warranted.

## Patients and methods

Patients with previously untreated HCC who were candidates for surgical resection, but not curable by resection or liver transplant were enrolled in Shanghai, China. Patients had a solitary lesion >5 cm in diameter, or 3 lesions with at least one > 3 cm, but no regional or distant metastatic disease. Patients had a good performance status, adequate blood counts and organ function. An autologous tumor cell (TC) line was established from the resected tumor. Irradiated autologous TC were incubated with autologous dendritic cells (DC) to create a patient-specific DC-TC product which was cryopreserved. Approximately eight weeks after one course of trans-arterial chemoembolization therapy (TACE), patients received three weekly subcutaneous injections of DC-TC suspended in 500 micrograms of sargramostim. Patients were monitored for toxicity for 8 weeks from the start of treatment.

## Results

HCC cell lines were successfully established within 5 weeks for 15/15 patients. Eight patients taking anti-viral medication for active HBV infection were treated (7 men, 1 woman) with ages ranging from 37 to 73 years. Each received the planned 3 injections, which were well tolerated. There were no significant changes in various parameters, including hepatic transaminases, bilirubin, prothrombin time, hepatitis B antigens, or viral DNA.

## Conclusion

Injections of autologous DC-TC in sargramostim were not associated with a worsening of hepatitis or other toxicities in HBV + HCC patients. This shows sufficient safety to justify a Phase II efficacy trial in similar patients.

